# Management of pericardial bleeding complications in percutaneous endo-epicardial catheter ablation for atrial fibrillation: A case for intrapericardial tranexamic acid?

**DOI:** 10.1016/j.hrcr.2022.09.004

**Published:** 2022-09-11

**Authors:** Johanna Tonko, Karthik Manoharan, Reshma Amin, John Silberbauer

**Affiliations:** ∗ Department of Cardiology, Royal Sussex County Hospital, Brighton and Sussex University Hospitals NHS Foundation Trust, Brighton, United Kingdom; † Institute for Cardiovascular Sciences, Centre for Translational Electrophysiology, University College London, London, United Kingdom

**Keywords:** Percutaneous epicardial AF catheter ablation, Epicardial bleeding, Pericardiocentesis, Sternotomy, Hemostasis, Systemic embolism, Tranexamic acid

## Introduction

Epicardial connections have been identified as an important cause for failed endocardial atrial fibrillation (AF) ablations. Percutaneous and/or surgical techniques for stand-alone epicardial or combined endo-epicardial ablations are increasingly used to overcome this limitation and achieve durable transmural lesions for sustained isolation of pulmonary veins and complete block across linear atrial lines, which has been shown to translate in a reduced AF / atrial tachycardia recurrence rate.^1^^,^^2^ This trend has been facilitated and promoted by the development of safer percutaneous subxiphoid epicardial access techniques, such as the carbon dioxide insufflation over intentional coronary vein exit, substantially reducing the risk of puncture-related complications^3^ and allowing to perform them on uninterrupted anticoagulation. Yet, despite the technical improvements for epicardial access, periprocedural risks specific to the hardware manipulation within the pericardial space remain, with experience from epicardial ventricular tachycardia procedures showing pericardial bleeding being one of the most common complications.

Here, we present a case of epicardial bleeding following percutaneous endo-epicardial AF ablation procedure on uninterrupted direct oral anticoagulants and the role of direct intrapericardial tranexamic acid application to achieve hemostasis.

## Case report

We report the case of a 43-year-old female patient with background of persistent AF diagnosed 6 years ago in the context of a thyrotoxicosis secondary to Graves disease and a history of excess alcohol consumption. After causative treatment of her thyroid dysfunction, a direct current cardioversion successfully converted her to sinus rhythm. Four years later, she re-presented with symptomatic persistent tachycardic AF and was noted to have a severely impaired ventricular function and severely dilated atria. Her thyroid hormone levels were within normal range and she underwent a first endocardial catheter ablation with acutely successful pulmonary vein isolation and posterior wall isolation (roof and floor line). Just after the blanking period she re-presented with a recurrence of persistent AF and decompensated heart failure. After preloading with amiodarone, a successful cardioversion was performed and she was able to maintain sinus rhythm while on continued amiodarone. Her left ventricular function improved to an ejection fraction of 45%.

Given her young age and presentation with heart failure, and in an attempt to wean her off amiodarone, she was referred for a redo AF ablation. To increase the chance of long-term rhythm control and high success rates in persistent AF with this procedure at our center, she was offered a combined percutaneous endo-epicardial approach on uninterrupted apixaban as per local protocol.

### Endo-epicardial AF ablation procedure

**Figure 1:**
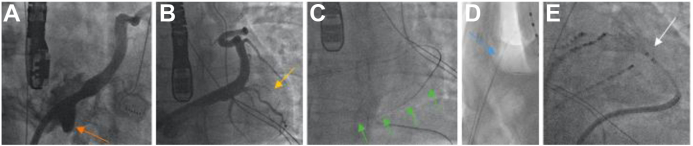
Epicardial access with CO_2_ insufflation technique over coronary vein exit in our patient. **A,B:** Coronary venogram in (**A**) right anterior oblique (large diverticula at coronary sinus os noted, *orange arrow*) and (**B**) left anterior oblique identifying suitable small side branch of a posterolateral branch (*yellow arrow*). **C:** Intentional coronary vein exit using a Confianza Pro wire (Asahi Intecc) (*green arrows*). **D:** Insufflation of 90 cc of CO_2_ over a micro-catheter and uncomplicated percutaneous subxiphoid puncture in 90° left lateral view on fluoroscopy (*blue arrow pointing at needle*). **E:** Insertion of steerable epicardial sheet in Seldinger technique and positioning of epicardial mapping catheter (*white arrow*) in pericardial space.

The procedure was performed using the EnSite X 3D EP mapping system (Abbott Laboratories, Abbott Park, IL) under general anesthesia and with transesophageal echocardiography. Routine antibiotic prophylaxis with single-shot cefazolin was administered. Triple right femoral venous access was gained under ultrasound guidance followed by epicardial access using the CO_2_ insufflation technique via a coronary vein exit, as described previously^4^ (Figure 1). The wire tracked freely in the pericardial space and also fluoroscopic images after CO_2_ insufflation did not raise any concerns for pericardial adhesions. Percutaneous epicardial puncture and insertion of a steerable sheath to the pericardial space was uncomplicated, with no relevant bleeding on aspiration and after attachment of a standard negative pressure drain to the side arm of the epicardial sheaths. The mapping catheter was placed in the oblique sinus on the posterior wall of the left atrium without meeting any resistance.

**Figure 2:**
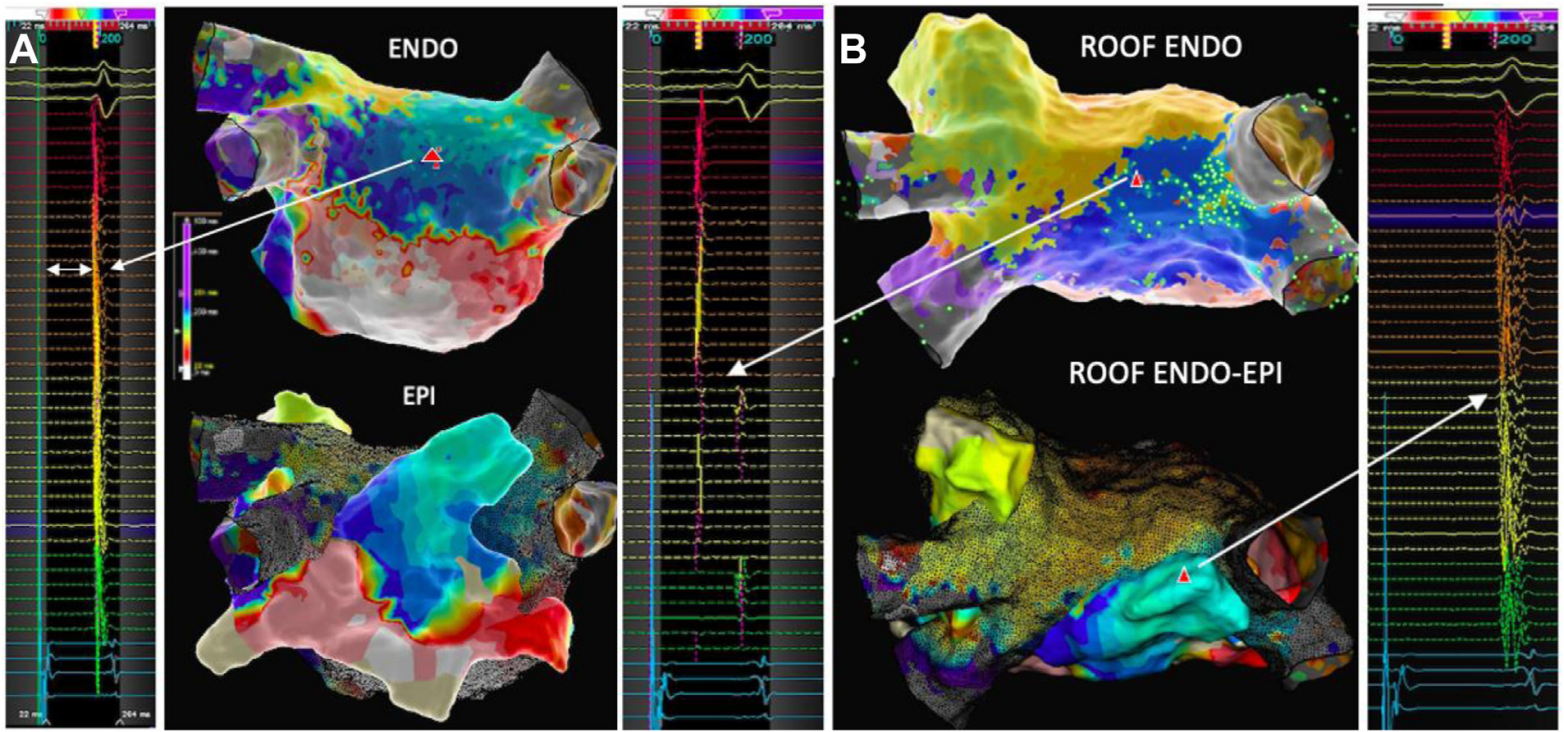
**A:** Posterior view on left atrial local activation time (LAT) map: Endocardial (top) and epicardial (bottom) LAT map with HD Grid (Abbott) during coronary sinus pacing, cycle length 600 ms. Posterior wall activated over slowly conducting epicardial connection on the roof, floor line blocked. Local electrogram in mid-posterior wall (*red triangle*) with significant delay of nearly 200 ms (*white arrow*) requiring extension of window of interest into QRS. **B:** View on left atrial roof (LAT). Top: Endocardial map with evidence of widely split double potentials (∼100 ms) on HD Grid splines positioned along roof line. Sparkles (*green bright dots*) indicate earliest activation of posterior wall occurs at right superior corner at site of insertion of septopulmonary bundle. Bottom: Epicardial map (full color) superimposed on endocardial map (black mesh) with evidence of long fractionated signals on opposing site to the earliest endocardial activation of the posterior box.

Subsequently a standard transesophageal echocardiography–guided transseptal puncture was performed and double endocardial left atrial access gained. Systemic anticoagulation with unfractionated heparin targeting an activated clotting time of >350 seconds was initiated, with no evidence of bleeding at this stage. An endocardial left atrial map with the Advisor HD Grid (Abbott) during pacing from the coronary sinus demonstrated a blocked floor line between the left and right inferior pulmonary veins but connected, yet very delayed, activation of the posterior wall (see local activation time map and electrogram in Figure 2A). Widely separated double potentials along the previous endocardial roof line were recorded and earliest activation of the posterior wall seemed to originate at the right superior posterior corner, highly suggestive of an epicardial connection over the septopulmonary bundle. Subsequent mapping of the epicardial surface of the left atrium in the oblique sinus confirmed this with evidence of long fractionated signals in the area opposing the earliest activation of the posterior wall (see endocardial and epicardial maps with respective electrograms in Figure 2B). Ablation at the corresponding endocardial and epicardial sites of suspected connection over the roof successfully isolated the posterior wall. An additional lesion set for an anterior mitral line from the roof line to the anterior mitral annulus was deployed and bidirectional block confirmed.

### Epicardial bleeding and management

**Figure 3:**
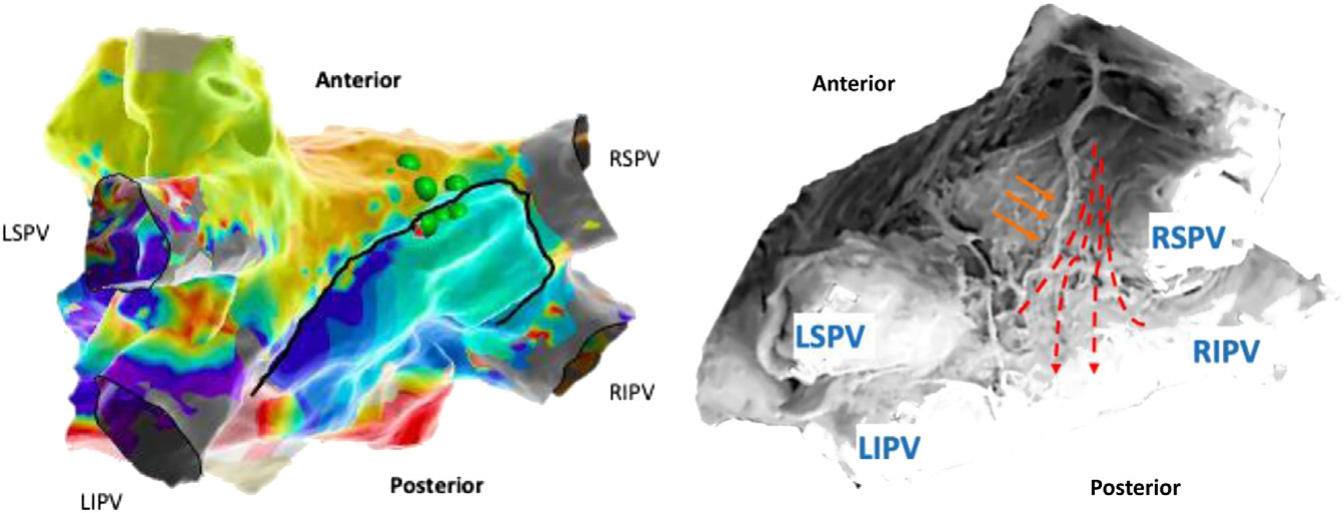
Left: Posterior view on endo-epicardial local activation time map (black line separating endo from superimposed epicardial map). Isolation of posterior box at right-sided roof line by endo-epicardial ablation (green points = ablation lesions). Start of bleed coinciding with positioning ablation catheter and delivering ablation lesions from epicardial site. Right: Anatomical model of left atrium demonstrating vascular network on roof/posterior wall (*orange arrows*). Dashed red lines indicating course of septopulmonary bundle, which was targeted for ablation. LIPV = left inferior pulmonary vein; LSPV = left superior pulmonary vein; RIPV = right inferior pulmonary vein; RSPV = right superior pulmonary vein.

After positioning of the ablation catheter in the oblique sinus and delivery of epicardial ablation (45 W) to the area of interest to consolidate block over the roof (Figure 3), new bleeding over the negative pressure drain attached to the sheath was noted. Following this, ablation was stopped, bidirectional block across the anterior mitral line and isolation of the posterior wall reconfirmed, and subsequently all catheters and sheaths removed from the left atrium. Heparin was reversed with protamine. Within the next 30 minutes around 250 mL of dark hemorrhagic fluid was drained by the redivac. The cardiac surgeons were informed and intravenous active prothrombin complex, tranexamic acid, and a platelet concentrate (following the results of an urgently sent blood count with platelets of 109 g/L) administered. Recombinant human Factor Xa (andexanet alfa) to reverse apixaban was not available at our hospital.

Even though the bleeding slowed down following these acute medical measures, there was an ongoing consistent drainage of around 1 mL/min over the next 2 hours. Given the comparatively small quantity of dark-colored blood and the temporal coincidence with manipulation on the posterior wall of the left atrium, we initially suspected a venous bleed from a vessel within that area, which would be difficult to reach for a surgical intervention. Based on the positive results for its topical use for bleeding in the context of cardiac surgery, direct intrapericardial application of tranexamic acid (2 g in 20 mL 0.9% saline) over the steerable sheath was injected, flushed with further 20 mL (to prevent clotting of the drain), and the drain clamped. Hemodynamically the patient remained stable throughout and the transesophageal echocardiography showed no evidence of accumulating, undrained fluid or visible clot formation within the pericardium. Subsequently the suction over the redivac re-established. After a further observation period of 1 hour with near-complete cessation of the bleed, the steerable sheath was switched to a standard 6F pericardial drain and the patient was extubated. Following extubation the patient was brought to a semi-sitting position to support static pressure on the back wall of the heart.

Thereafter no relevant bleeding/drainage was observed and serial echocardiography excluded any reaccumulating fluid in the pericardial space. The drain was removed the following day and oral anticoagulation re-established in the evening thereafter. The patient reported some pericarditic pain, which was well controlled with oral analgesia. After an otherwise uneventful clinical course the patient was discharged on day 2 after her procedure.

At 3 months follow-up she remained in sinus rhythm. Her initial pericarditic pain had settled completely.

## Discussion

Epicardial connections are a recognized important cause for failure of endocardial-only ablations for pulmonary vein isolation^5^ as well as for blocking linear lesions.^6^ This has been explained by the thicker myocardium in these areas as well as fat interposition between the subendocardial and subepicardial myocardium hampering transmural lesion formation.^6^ Followed by initial encouraging case reports and small case series,^7^, ^8^, ^9^ in recent years percutaneous combined endo-epicardial AF catheter ablation by interventional electrophysiologists has been evaluated in larger cohorts and confirmed to yield similarly encouraging results compared to surgical and hybrid endo-epicardial AF ablations with a low periprocedural morbidity.^2^^,^^10^^,^^11^

Pericardial complications remain the second most common complication (after femoral vascular access complications) in patients undergoing complex electrophysiological procedures. This may be secondary to direct mechanical trauma by catheter/sheath manipulation overheating during radiofrequency energy delivery or misdirected transseptal punctures for the endocardial part. For endocardial AF ablations only, reported incidence of cardiac tamponade range between 0.2% and 5%.^12^ The largest percutaneous endo-epicardial AF ablation series up to today report a pericardial bleeding risk of 2% and freedom from AF was 73% after 24 months in a mixed population of paroxysmal, persistent, and longstanding persistent AF.^2^ Pericardial bleeding complications in the recent CONVERGE hybrid endo-epi AF ablation trial using surgical transdiaphragmatic/subxiphoid approach occurred in 4.9% and freedom of AF was 71% at 12 months for persistent and longstanding persistent AF.^13^

### Management of pericardial complications during endo-epicardial AF procedures

In combined endo-epicardial procedure, commonly a drain or sheath attached to a negative pressure drain is present within the pericardial space and first signs of bleeding are usually manifest by an increased drainage of hemorrhagic fluid. In case of evidence of a significant arterial bleed over the drain (rapid drainage of large quantity of bright red blood), surgical exploration and intervention is required, as medical measures are unlikely to be successful without active closure of the source. Yet, a significant number of iatrogenic pericardial bleeds during ablation present in a gray area where decision-making about conservative vs surgical treatment may not be straightforward. Controversies exist particularly in relation to timing of reversal of heparin and active administration of prothrombotic medication. Even though the primary aim is to control the causal bleeding source and prevent a cardiac tamponade, any measure must be balanced against the risk of endocardial thrombus apposition on the recent ablation lesions and risk of stroke. To prevent systemic prothrombotic states and minimize aforementioned risk of thrombus formations, local hemostatic measures, whenever feasible, are to be preferred. Direct intrapericardial application of the antifibrinolytic agent tranexamic acid, a synthetic drug derived from lysin binding to plasminogen and thereby reducing its conversion to plasmin, may be a useful adjunct measure to support local hemostasis without systemic effects. Human pericardium contains high levels of tissue plasminogen activator, increasing local fibrinolytic activity to maintain fluidity under normal conditions, and fibrinolytic activity may be further enhanced by tissue manipulation.^14^ A recent meta-analysis suggested that intrapericardial use of tranexamic acid in patients undergoing cardiac surgery can decrease the postoperative bleeding risk without increasing the risk of thromboembolic events or seizures.^15^

Experience with intrapericardial tranexamic acid in percutaneous epicardial procedure and its application over the drain is sparse. To our knowledge, our case is the first to report its use for percutaneous epicardial procedures with, here, successful hemostasis, averting the need of a surgical intervention. Previous large studies from surgical patients have shown that it is feasible to dilute it also in small amounts of fluid (1 g / 10 mL saline)^16^ and studies for dental procedures using oral rinse with tranexamic acid for as short as 2 minutes can help reduce bleeding.^17^

## Conclusion

Percutaneous endo-epicardial AF ablations are efficient procedures for achieving transmural linear lesions across the left atrial wall. Safer and more sophisticated techniques in epicardial access have allowed to substantially reduce epicardial access–related complications. Pericardial bleeding events arising at a later stage in the procedure after having delivered endocardial lesions while on full heparinization pose a particular medical dilemma. Local measures to promote hemostasis are preferable, whenever feasible, to avoid generating systemic prothrombotic states with increased risk of systemic thromboembolism. Intrapericardial tranexamic acid may be a useful adjunct for a subset of these cases. Yet, experience with this application is sparse, and in our case report it remained open how much absolute additive benefit local tranexamic acid provided in eventually achieving hemostasis. The risk of clotting of the pericardial drain itself, if not appropriately flushed after application of tranexamic acid, is a potential concern. Also there remains a debate whether topical application may increase formation of adhesions or, owing to its anti-inflammatory effects, actually do the opposite and inhibit their formation.

**Key Teaching Points**

• Catheter and sheath manipulation within the pericardial space and delivery of radiofrequency ablation lesions carry a recognized risk for bleeding and hemopericardium, requiring individualized management strategies depending on suspected source of bleed, quantity, and hemodynamic stability.
• Management of bleeding events in the context of simultaneous endo-epicardial ablation procedures poses a particular challenge owing to the delicate balance between achieving haemostasis while avoiding systemic prothrombotic states facilitating thrombus apposition on endocardial lesions with risk of embolization/stroke.
• Intrapericardial tranexamic acid can be successfully applied as an adjunctive therapy for promoting local hemostasis in a subset of iatrogenic epicardial bleeds without increasing risk of endocardial thrombus formation.
• Early involvement of the cardiac surgical team is always advisable for multidisciplinary decision-making and prevention of unnecessary delays in case of ongoing uncontrolled bleeding requiring surgical intervention.
